# Agonism of PIEZO1 prevents aggravated periodontitis with traumatic occlusion via MAPK signaling pathway

**DOI:** 10.1016/j.isci.2025.113688

**Published:** 2025-10-08

**Authors:** Xia Wang, Binqing Xie, Ye Guo, Haiyin Wan, Xianyi He, Junliang Chen, Yun He

**Affiliations:** 1 Department of Oral and Maxillofacial Surgery, the Affiliated Stomatological Hospital, Southwest Medical University, Luzhou Sichuan, China; 2 Luzhou Key Laboratory of Oral and Maxillofacial Reconstruction and Regeneration, Luzhou Sichuan, China; 3 Department of General Dentistry, The Affiliated Stomatological Hospital, Southwest Medical University, Luzhou, Sichuan, China

**Keywords:** Mechanobiology, Cell biology

## Abstract

Periodontitis is a chronic inflammatory disease causing alveolar bone loss and tooth damage. Traumatic occlusion (TO) exacerbates its progression, although the process is mechanistically unclear. This study investigated the role of the mechanosensitive ion channel PIEZO1 in TO-aggravated periodontal destruction. In a rat model combining periodontitis and TO, PIEZO1 expression decreased, with increased inflammation and impaired osteogenesis. Yoda1 treatment alleviated periodontal inflammation and bone loss, demonstrating its therapeutic potential. *In vitro*, mechanical stress and lipopolysaccharide (LPS) synergistically downregulated PIEZO1 in human periodontal ligament fibroblasts (hPDLFs), reducing osteogenic potential and elevating pro-inflammatory responses. Transcriptomics identified mitogen-activated protein kinase (MAPK) pathway inhibition as a key downstream effect. Yoda1-mediated activation restored Extracellular Signal-regulated Kinase (ERK), Jun N-terminal Kinase (JNK), and p38 phosphorylation; promoted osteogenesis; and suppressed inflammatory cytokines. Overall, TO exacerbates periodontitis by suppressing PIEZO1-MAPK signaling, and PIEZO1 activation may protect periodontal tissues from mechanical and inflammatory damage.

## Highlights

• PIEZO1 downregulation by traumatic occlusion exacerbates periodontitis progression
• Yoda1-mediated PIEZO1 activation reduces inflammatory bone loss in a rat model
• Compressive stress and LPS suppress PIEZO1-MAPK osteogenic signaling in hPDLFs
• PIEZO1 activation restores MAPK activity and enhances periodontal tissue repair

## Graphical abstract

**Figure:**
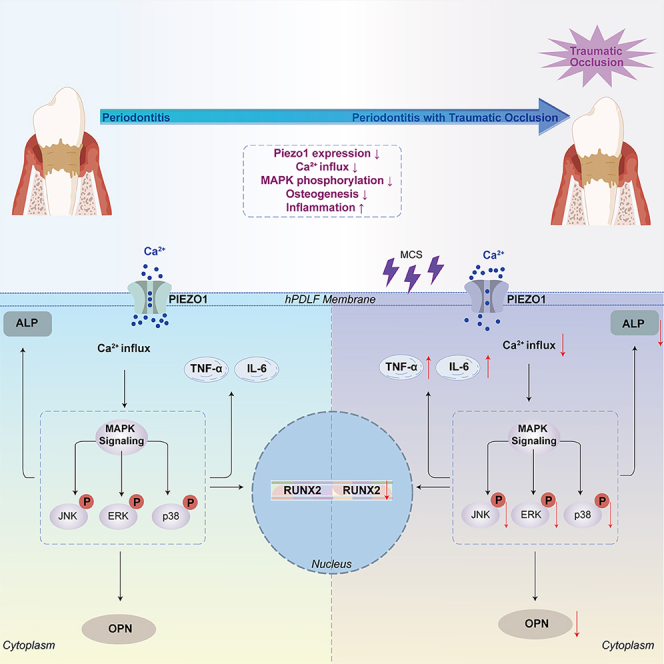

## Introduction

Periodontitis (PD) is a multifactorial infectious and inflammatory disease characterized by the progressive loss of periodontal attachment, alveolar bone resorption, and ultimately tooth mobility and loss.^1^ Its onset and progression are influenced by various factors, including microbial infection, host immune responses, and environmental conditions.^2^^,^^3^^,^^4^

Traumatic occlusion (TO) refers to pathological alterations in the masticatory system resulting from abnormal occlusal contacts or excessive occlusal forces.^5^ The periodontal tissue response to isolated TO typically progresses through three distinct phases: trauma, repair, and adaptation/remodeling.^6^ However, when TO co-exists with PD, it can exacerbate alveolar bone destruction and accelerate periodontal breakdown and tooth loss.^7^^,^^8^ Despite this clinical observation, the underlying mechanisms by which TO aggravates PD remain poorly understood.

The ability of the periodontium to sense and respond to occlusal forces is closely linked to the presence of mechanosensitive ion channels within periodontal tissues and cells.^9^ Among these, the piezo type mechanosensitive ion channel component (PIEZO) family represents an evolutionarily conserved group of ion channels that play a central role in mechanotransduction.^10^ PIEZO1, in particular, is critically involved in sensing external mechanical forces and regulating tissue and organ development.^11^ Human periodontal ligament fibroblasts (hPDLFs) are known to be mechanosensitive, and previous studies have shown that PIEZO1 contributes to their response to mechanical stimulation.^12^^,^^13^ Based on these findings, we hypothesize that PIEZO1 mediates the mechanosensory transduction of traumatic occlusal forces in periodontal tissues.

Mitogen-activated protein kinases (MAPKs) are a family of highly conserved protein kinases widely expressed in most cell types, which function to transduce extracellular signals into intracellular and nuclear responses.^14^ MAPKs are intracellular signaling molecules activated in response to various inflammatory mediators^15^ and play critical regulatory roles in the pathogenesis of periodontitis.^16^ In hPDLFs, inflammatory stimuli have been shown to suppress MAPK pathway activation.^17^ Moreover, emerging evidence suggests a functional association between MAPK signaling and the mechanosensitive ion channel PIEZO1.^18^ In human periodontal cells, PIEZO1 has been implicated in mechanotransduction by transmitting mechanical signals via the ERK1/2 pathway.^19^

Based on these findings, we hypothesized that PIEZO1 contributes to the exacerbation of periodontitis under traumatic occlusion via modulation of MAPK signaling. To test this, we established an *in vivo* Sprague-Dawley (SD) rat model of periodontitis combined with traumatic occlusion to evaluate the temporal effects of mechanical trauma on periodontal destruction, PIEZO1 expression, and MAPK signaling. In parallel, we applied mechanical stress and inflammatory stimulation to hPDLFs *in vitro* to investigate the underlying molecular mechanisms of PIEZO1-MAPK signaling in this dual-stress context.

## Results

### TO aggravated periodontal tissue inflammation and bone loss in rats with periodontitis

**Figure 1:**
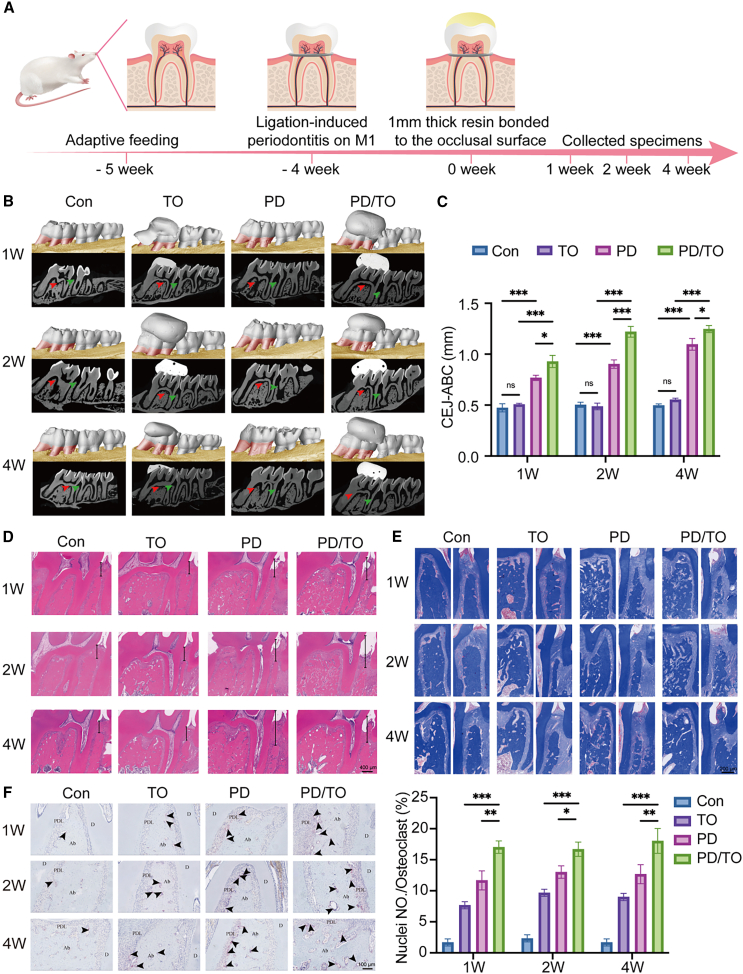
Traumatic occlusion aggravated periodontal tissue inflammation and bone loss in rats with periodontitis (A) Schematic representation of rat models for periodontitis and traumatic occlusion experiments. (B) Three-dimensional reconstructions and two-dimensional sagittal plane images obtained through Micro-CT. Red arrows: M1 root bifurcation; green arrows: interval between the distal and mesial alveolar regions of M1. (C) Quantitative assessment of the distance of CEJ-ABC at the distal root. (D) H&E staining of M1 and periodontal tissue. Scale bar: 400 μm. (E) Masson’s trichrome staining of periodontal tissue in the distal and root furcation regions of M1. Scale bar: 200 μm. (F) TRAP staining of the M1 root furcation region. Scale bar: 100 μm. Representative histological images from a single representative sample in the same group. Consecutive sections were subjected to H&E staining (D), Masson’s trichrome staining (E), and TRAP staining (F). PDL, periodontal ligament; D, dentin; Ab, alveolar bone. Black arrow: osteoclast. Statistical significance was tested with Kruskal-Wallis test followed by Dunn’s *post ho*c test. ∗*p* < 0.05, ∗∗*p* < 0.01, and ∗∗∗*p* < 0.001; ns, not significant (*p* > 0.05). Error bars represent mean ± SD (*n* = 6 biological replicates).

Animal models of TO, PD, and combined PD/TO were successfully established in SD rats. After three-dimensional reconstruction using Micro-CT (Figure 1B), the distance of Cemento-enamel junction-Alveolar bone crest (CEJ-ABC) at the distal root was measured and quantified (Figure 1C). The results showed that, compared to the Control (Con) group, CEJ-ABC was significantly increased in the PD and PD/TO groups at 1, 2, and 4 weeks (*p* < 0.05). We also found that alveolar bone height was significantly decreased in the PD/TO group compared to the PD group at all time points, with the most significant difference (*p* < 0.01) observed at 2 weeks.

Histopathological analysis revealed characteristic features of periodontitis including significant conjunctive epithelial retraction and obvious resorption of alveolar bone. As shown in the H&E-stained sections (Figure 1D), compared with the Con group, the TO group exhibited no significant loss of soft or hard tissue, except for the loosening of the alveolar bone in the root bifurcation area, while the conjunctive epithelial retraction and resorption of alveolar bone were observed in the TO and PD/TO groups at 1, 2, and 4 weeks. Notably, the PD/TO group exhibited time-dependent exacerbation of periodontal tissue destruction, compared to PD alone.

Masson staining results (Figure 1E) further demonstrated disrupted collagen organization in the distal root and bifurcation regions of the first molar (M1) in the TO, PD, and PD/TO groups compared to the Con group. Collagen fibers appeared disorganized and loosely arranged, indicating compromised integrity of the periodontal ligament (PDL).

The tartrate-resistant acid phosphatase (TRAP) staining results, shown in Figure 1F, revealed active osteoclasts in the root bifurcation region of the TO, PD, and PD/TO groups. As time progressed, the number of osteoclasts gradually increased in all groups. Quantitative analysis showed a significant increase in the PD/TO group compared to both the TO and PD groups (*p* < 0.05).

### PIEZO1 plays a role in the aggravation of periodontitis induced by traumatic occlusion

**Figure 2:**
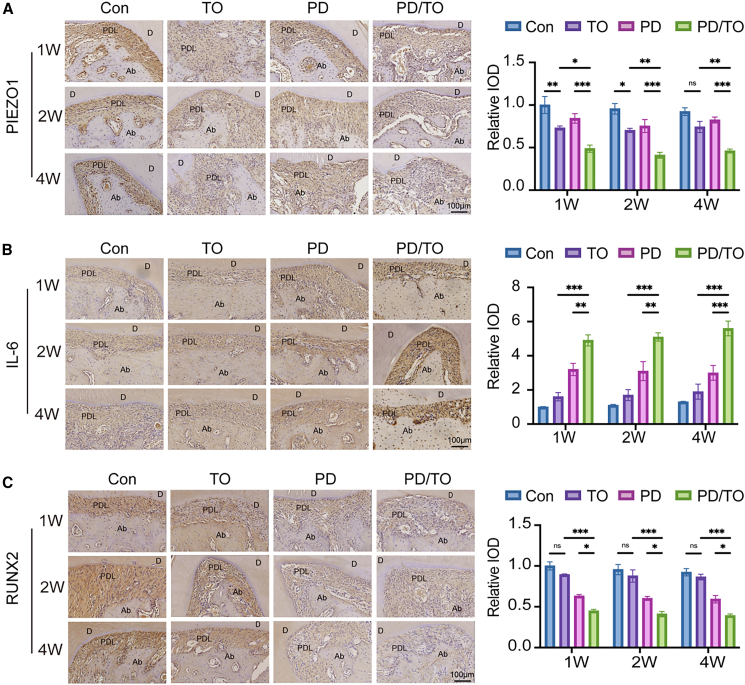
PIEZO1 plays a role in the aggravation of periodontitis induced by traumatic occlusion (A) Representative picture of IHC staining and semi-quantitative analysis of PIEZO1. Scale bar: 100 μm. (B) Representative picture of IHC staining and semi-quantitative analysis of RUNX2. Scale bar: 100 μm. (C) Representative picture of IHC staining and semi-quantitative analysis of IL-6. Representative histological images from a single representative sample in the same group. Scale bar: 100 μm. Consecutive sections were subjected to IHC staining of PIEZO1 (A), IHC staining of RUNX2 (B), and IHC staining of IL-6 (C). PDL, periodontal ligament; D, dentin; Ab, alveolar bone. Statistical significance was tested with Kruskal-Wallis test followed by Dunn’s *post hoc* test. ∗*p* < 0.05, ∗∗*p* < 0.01, and ∗∗∗*p* < 0.001; ns, not significant (*p* > 0.05). Error bars represent mean ± SD (*n* = 6 biological replicates).

Micro-CT and histological staining results indicated that TO exacerbates the inflammatory response in periodontitis. To explore the mechanistic role of PIEZO1 in TO-associated periodontitis progression, we performed immunohistochemistry (IHC) staining to analyze the expression of PIEZO1, interleukin (IL)-6, and runt-related transcription factor 2 (RUNX2) in PDL tissue. Semi-quantitative analyses results demonstrated that PIEZO1 expression was significantly reduced in the PD/TO group compared with other groups (Figure 2A, *p* < 0.05). Interestingly, PIEZO1 downregulation was observed in both the TO and PD/TO groups, whereas no significant difference was detected in the PD group versus the Con group. We speculated that TO is the primary factor contributing to the decrease in PIEZO1 expression.

The expression of IL-6 in the PD/TO group was markedly elevated compared to the other groups (Figure 2B, *p* < 0.01). Conversely, RUNX2 levels exhibited an inverse correlation with IL-6. RUNX2 expression was highest in the Con group and lowest in the PD/TO group. Compared to the Con, TO, and PD groups, RUNX2 expression was significantly decreased in the PD/TO group (Figure 2C, *p* < 0.05).

The results demonstrated that TO led to a significant reduction in PIEZO1 expression in the PDL region. Moreover, when TO group combined with PD group, the expression of PIEZO1 was further reduced. Furthermore, TO was found to exacerbate inflammation and enhance osteoclastic activity, contributing to the progression of periodontitis. Collectively, TO exacerbated periodontal inflammation and bone resorption while disrupting PIEZO1-mediated mechanotransduction.

### Yoda1 treatment attenuates periodontal tissue destruction and osteoclast activity in the PD/TO rat model

**Figure 3:**
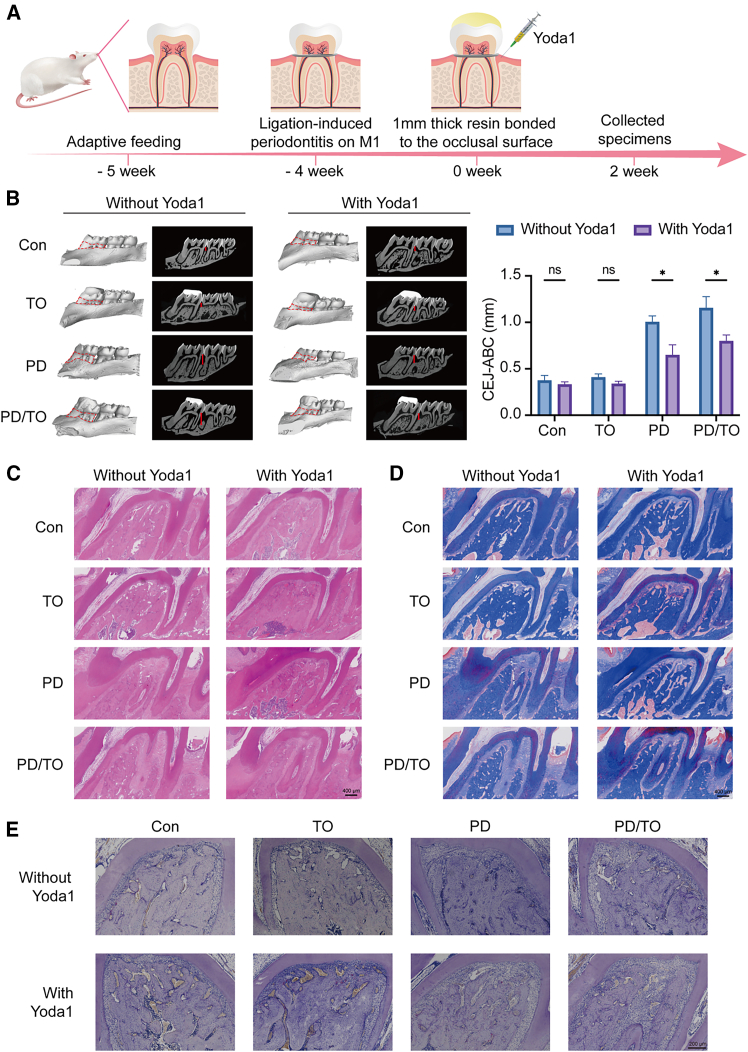
Yoda1 treatment attenuates periodontal tissue destruction and osteoclast activity in the PD/TO rat model (A) Schematic diagram of the animal experimental design, including model establishment and Yoda1 administration. (B) Micro-CT images showing alveolar bone loss around maxillary first molars. Quantification of CEJ-ABC was performed. (C) H&E staining of periodontal tissues showing inflammatory cell infiltration and structural preservation. Scale bar: 400 μm. (D) Masson’s trichrome staining showing collagen fiber distribution and organization. Scale bar: 400 μm. (E) TRAP staining showing osteoclasts along the alveolar bone surface. Scale bar: 200 μm. Representative histological images from a single representative sample in the same group. Consecutive sections were subjected to H&E staining (C), Masson’s trichrome staining (D), and TRAP staining (E). Statistical significance was tested with Mann-Whitney U test. ∗*p* < 0.05, ∗∗*p* < 0.01, and ∗∗∗*p* < 0.001; ns, not significant (*p* > 0.05). Error bars represent mean ± SD (*n* = 6 biological replicates).

To evaluate the therapeutic effect of PIEZO1 activation *in vivo*, we assessed alveolar bone morphology and periodontal tissue structure using Micro-CT and histological staining. As shown in Figure 3B, Micro-CT analysis revealed that rats in the PD/TO + Yoda1 group exhibited significantly reduced alveolar bone loss compared to the untreated PD/TO group. Specifically, quantitative measurement of CEJ-ABC showed a marked decrease in the Yoda1-treated group, indicating preservation of alveolar bone height.

Histological evaluation further supported these findings. H&E staining (Figure 3C) demonstrated decreased inflammatory cell infiltration and better preservation of periodontal architecture in Yoda1-treated rats. Similarly, Masson’s staining (Figure 3D) showed improved collagen fiber organization and reduced connective tissue degradation.

In addition, TRAP staining (Figure 3E) revealed a significant reduction in TRAP-positive osteoclasts along the alveolar bone surface in the Yoda1-treated group, indicating suppressed osteoclastic activity and bone resorption.

Collectively, these results indicate that local PIEZO1 activation via Yoda1 effectively alleviates periodontal inflammation, preserves tissue integrity, and reduces osteoclast-mediated bone loss in rats with periodontitis aggravated by traumatic occlusion.

### Yoda1 activates the MAPK pathway by promoting the expression of PIEZO1

Previous studies demonstrated that TO accelerated periodontitis progression by inhibiting PIEZO1 expression, whereas Yoda1 attenuated alveolar bone loss and inflammation. To further investigate the correlation between PIEZO1 and the MAPK signaling pathway, we assessed protein expression using immunohistofluorescence (IHF) staining for PIEZO1 and phosphorylated MAPK signaling pathway components (phosphorylated Jun N-terminal Kinase [p-JNK] and phosphorylated Extracellular Signal-Regulated Kinase [p-ERK]) and IHC staining for the osteogenic transcription factor RUNX2.

**Figure 4:**
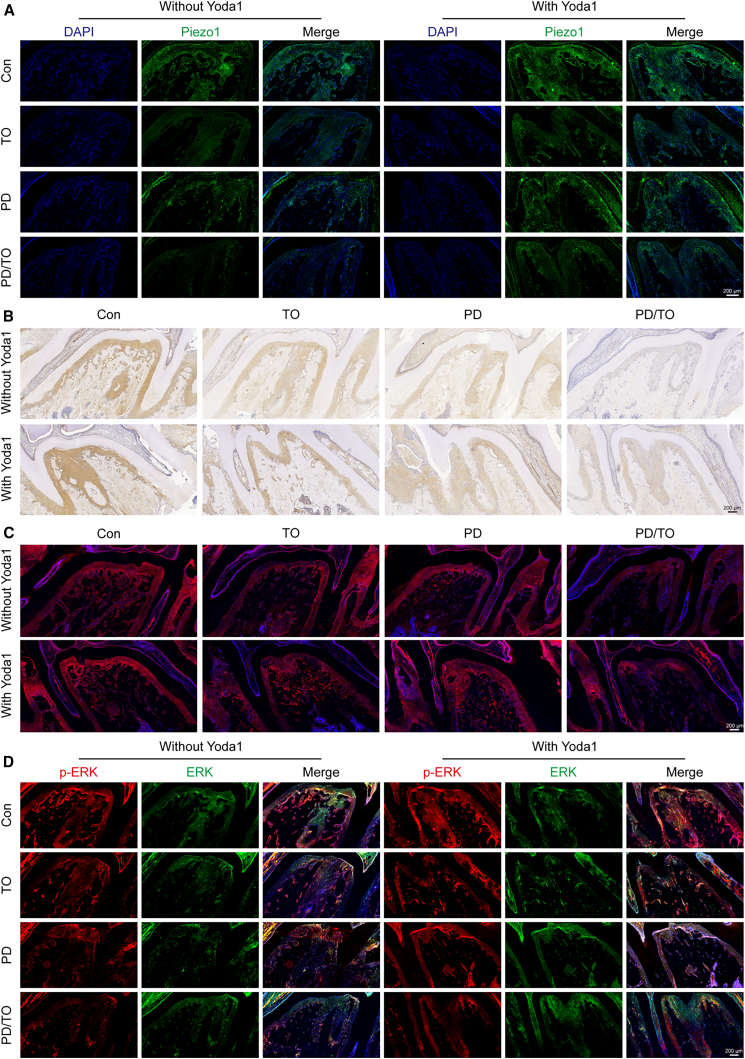
Yoda1 activates the MAPK pathway by promoting the expression of PIEZO1 (A) Representative picture of IHF staining showing expression of PIEZO1 in the periodontal ligament. (B) Representative picture of IHC staining showing expression of RUNX2 in the periodontal ligament. (C) Representative picture of IHF staining showing expression of p-JNK in the periodontal ligament. (D) Representative picture of IHF staining showing expression of ERK and p-ERK in the periodontal ligament. Scale bar: 200 μm. Representative histological images from a single representative sample in the same group. Consecutive sections were subjected to IHF staining of PIEZO1 (A), IHC staining of RUNX2 (B), IHF staining of p-JNK (C), and IHF staining of ERK and p-ERK (D) (*n* = 6 biological replicates).

As shown in Figure 4A, Yoda1 treatment significantly upregulated PIEZO1 expression. Concurrently, RUNX2 expression was also markedly elevated (Figure 4B). Figures 4C and 4D revealed that p-JNK and p-ERK expression were suppressed in both the TO and PD/TO groups, with maximal inhibition observed in the PD/TO group. Following Yoda1 administration, p-JNK and p-ERK levels increased across all groups, with the PD/TO group exhibiting the most significant upregulation.

These results indicate that PIEZO1 expression functionally correlates with MAPK signaling pathway activity. Specifically, PIEZO1 activation promotes phosphorylation of the key MAPK effectors—JNK and ERK. This PIEZO1-MAPK axis contributes to the inhibition of alveolar bone resorption in traumatic occlusal periodontitis.

### Isolation and characterization of hPDLFs

**Figure 5:**
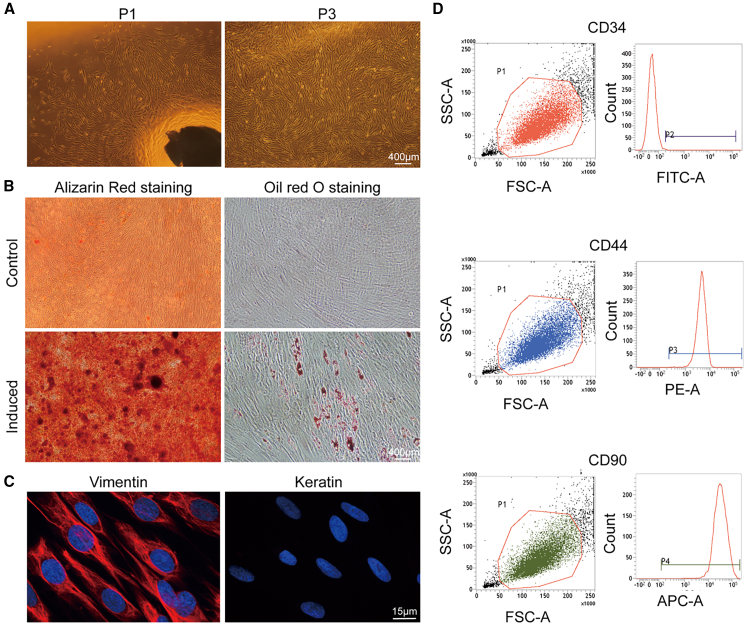
Isolation and characterization of hPDLFs (A) Primary and passaged culture of hPDLFs. Scale bar: 400 μm. (B) Alizarin red S and oil red O staining showed that hPDLFs had osteogenic and lipogenic differentiation capacity. Scale bar: 400 μm. (C) ICF staining showed positive expression of vimentin and negative expression of keratin in hPDLFs. Scale bar: 15 μm. (D) Flow cytometry detected positive expression of surface antigens CD44 and CD90 and negative expression of CD34 in hPDLFs.

HPDLFs were isolated from the PDL of extracted teeth. Generally, the cells exhibited a radial, vortex-like arrangement with a thin, elongated spindle-shaped morphology (Figure 5A). Following 3 weeks of osteogenic induction, alizarin red staining confirmed the presence of red-colored mineral deposits (Figure 5B). Furthermore, oil red O staining revealed lipid droplet formation after 3 weeks of adipogenic induction (Figure 5B), further supporting the multipotent differentiation capacity of hPDLFs. immunocytofluorescence (ICF) staining indicated that hPDLFs were strongly positive for vimentin and negative for keratin (Figure 5C), confirming the mesenchymal origin of hPDLFs. Flow cytometric analysis of surface marker expression revealed high positivity for CD44 and CD90, while CD34 was negative, consistent with the phenotype of mesenchymal stem cells (Figure 5D). The subsequent *in vitro* experiments were performed using hPDLFs at passage 3 to ensure consistency and reproducibility.

### MCS loading on hPDLFs

**Figure 6:**
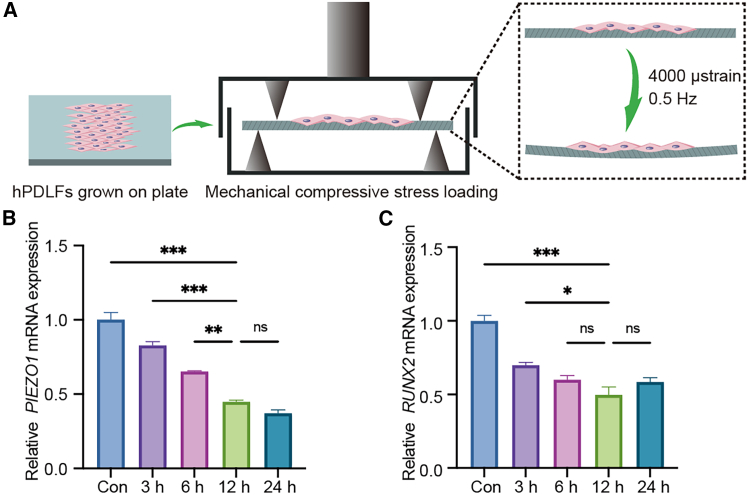
MCS loading on hPDLFs (A) Schematic of MCS loading on hPDLFs with a magnitude of 4,000 μstrain and a frequency of 0.5 Hz. The expression of *PIEZO1* (B) and *RUNX2* (C) detected by RT-qPCR and quantitative analysis. Statistical significance was tested with Kruskal-Wallis test followed by Dunn’s *post hoc* test. ∗*p* < 0.05, ∗∗*p* < 0.01, and ∗∗∗*p* < 0.001; ns, not significant (*p* > 0.05). Error bars represent mean ± SD (*n* = 3 biological replicates).

A four-point bending system was used to apply mechanical compressive stress (MCS) to hPDLFs and evaluate its effect on PIEZO1 expression. The experimental setup for MCS loading was shown in Figure 6A. Quantitative reverse-transcription PCR (RT-qPCR) analysis indicated that the expression of *PIEZO1* and *RUNX2* decreased with prolonged loading time, reaching their lowest levels at 12 h. Based on these findings, a 12-h MCS duration was selected for subsequent experiments to ensure optimal experimental conditions (Figure 6B).

### The effect of MCS loading on the expression of PIEZO1, osteogenic factors, and inflammatory factors in both normal and inflammatory hPDLFs

MCS loading was applied to both normal and lipopolysaccharide (LPS)-induced inflammatory hPDLFs, followed by evaluation of PIEZO1, osteogenic markers (ALP, RUNX2, and osteopontin [OPN]), and inflammatory cytokines (IL-6 and tumor necrosis factor alpha [TNF]).

**Figure 7:**
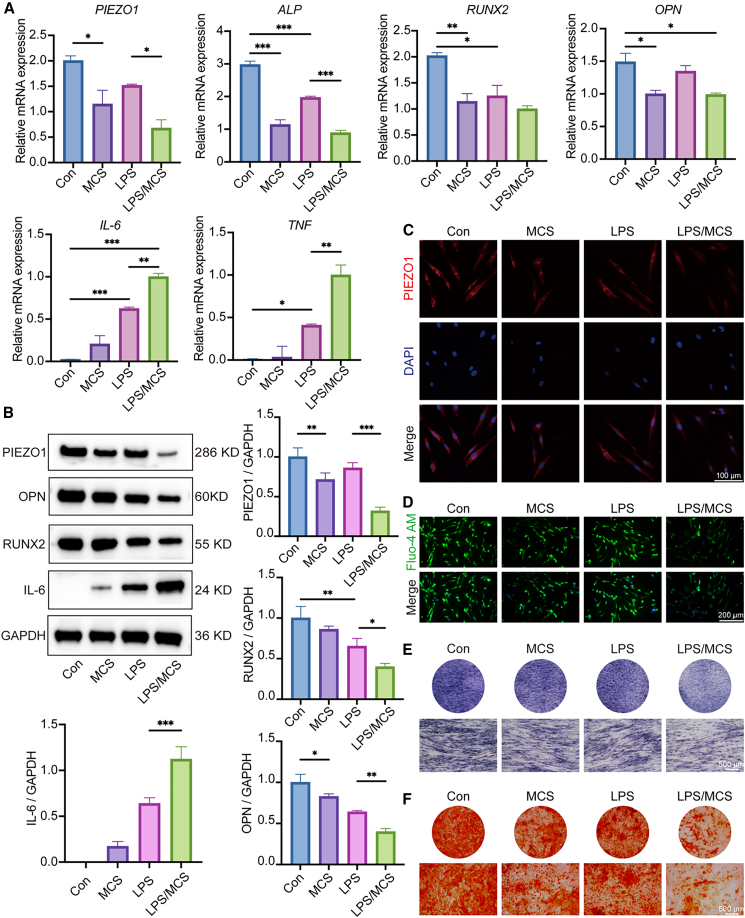
The effect of MCS loading on the expression of PIEZO1, osteogenic factors, and inflammatory factors in both normal and inflammatory hPDLFs (A) Gene expression of *PIEZO1*, *RUNX2*, *OPN*, *ALP*, *IL-6*, and *TNF* was assessed by RT-qPCR. (B) Protein expression of PIEZO1, RUNX2, OPN, and IL-6 was detected by WB, and the semi-quantitative analysis was performed using ImageJ. (C) Expression of PIEZO1 analyzed by ICF. Scale bar: 100 μm. (D) Expression of Ca^2+^ in hPDLFs. Scale bar: 200 μm. (E) ALP staining of hPDLFs with images captured under an inverted microscope. Scale bar: 500 μm. (F) ARS staining of hPDLFs with images captured under an inverted microscope. Scale bar: 500 μm. Statistical significance was tested with Kruskal-Wallis test followed by Dunn’s *post hoc* test. ∗*p* < 0.05, ∗∗*p* < 0.01, and ∗∗∗*p* < 0.001; ns, not significant (*p* > 0.05). Error bars represent mean ± SD (*n* = 3 biological replicates).

The results of RT-qPCR showed significant downregulation of *PIEZO1* in the MCS group compared to the Con group. Additionally, the expression of *PIEZO1* in the LPS/MCS group was significantly decreased (*p* < 0.05) compared to the LPS group (Figure 7A). Notably, both MCS and LPS/MCS groups exhibited markedly reduced expression of *PIEZO1*, *ALP*, *RUNX2*, and *OPN* relative to the Con group (*p* < 0.05) (Figure 7A). However, the expressions of *PIEZO1* and *OPN* were not significantly altered in the LPS group (*p* > 0.05) (Figure 7A). Compared with the Con group, the expressions of *IL-6* and *TNF* were significantly increased (*p* < 0.05) in both the LPS and LPS/MCS groups, but no significant increase in *IL-6* and *TNF* was observed in the MCS group (*p* > 0.05). Critically, the LPS/MCS group displayed significantly lower *PIEZO1* and *ALP* levels and higher *IL-6* and *TNF* expression than the LPS group (*p* < 0.05) (Figure 7A).

Western blot (WB) results (Figure 7B) demonstrated pronounced reductions in PIEZO1, RUNX2, and OPN protein levels in the MCS group, LPS group, and LPS/MCS group compared with the Con group (*p* < 0.001). Compared with the LPS group, the protein expression levels of PIEZO1, RUNX2, and OPN were significantly lower in the LPS/MCS group, along with increased IL-6 protein levels in the LPS/MCS group.

Consistently, ICF staining for PIEZO1 was consistent with those observed in RT-qPCR and WB analyses (Figure 7C). The red fluorescence was strongest in the Con group and weakest in the LPS/MCS group. Additionally, the expression of PIEZO1 in the LPS/MCS group was significantly lower compared to the LPS group. In addition, calcium ion fluorescence staining showed the strongest intracellular Ca^2+^ signal in the control group, while signal intensity decreased in the MCS and LPS groups and was lowest in the LPS/MCS group (Figure 7D), indicating impaired PIEZO1-mediated calcium influx.

To assess osteogenic function, Alkaline Phosphatase (ALP) staining (Figure 7E) and Alizarin Red S (ARS) staining (Figure 7F) were performed. The LPS/MCS group exhibited the weakest ALP activity and the fewest and faintest mineralized nodules, highlighting compromised osteogenic potential under combined mechanical and inflammatory conditions.

### mRNA sequencing analysis to explore the mechanism by which MCS affects osteogenesis in inflammatory hPDLFs

To clarify the potential mechanisms underlying MCS-mediated regulation of osteogenesis and inflammation in hPDLFs, we conducted mRNA sequencing analysis on samples from all four groups.

**Figure 8:**
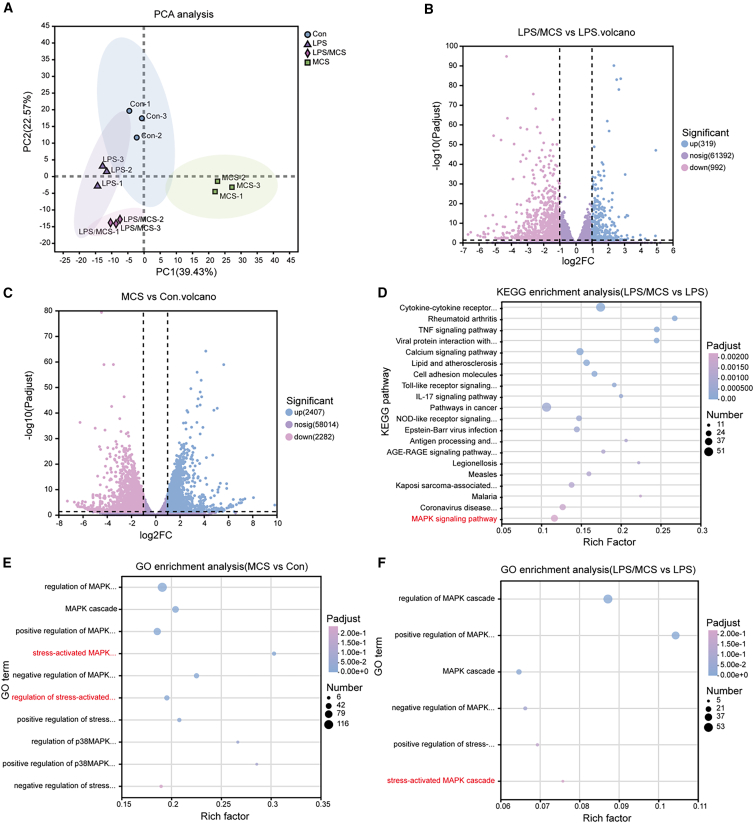
mRNA sequencing analysis to explore the mechanism by which MCS affects osteogenesis in inflammatory hPDLFs (A) PCA analysis among the Con group, the MCS group, the LPS group, and the LPS/MCS group. (B) The volcano of DEGs between the LPS group and the Con group. (C) The volcano of DEGs between the LPS/MCS group and the LPS group. (D) KEGG enrichment analysis of DEGs between the LPS/MCS group and the LPS group. (E) GO enrichment analysis of the MAPK signaling pathway between the LPS group and the Con group. (F) GO enrichment analysis of the MAPK signaling pathway between the LPS/MCS and the LPS group (*n* = 3 biological replicates).

As illustrated in Figure 8A, principal-component analysis (PCA) demonstrated distinct clustering among the four groups, highlighting distinct gene expression profiles. Indeed, differential expression genes (DEGs) analysis identified 2,407 upregulated genes and 2,282 downregulated genes in the MCS group versus the Con group, as well as 319 upregulated genes and 992 downregulated genes in the LPS/MCS group compared to the LPS group (Figures 8B and 8C). To further investigate the biological process involved in these DEGs, Kyoto Encyclopedia of Genes and Genomes (KEGG) analysis was performed. The results demonstrated significant enrichment of the MAPK signaling pathway in the LPS/MCS group (Figure 8D). Gene Ontology (GO) analysis further confirmed that DEGs were closely associated with MAPK signaling pathways, particularly the stress-activated MAPK cascade (Figures 8E and 8F). In conclusion, transcriptome sequencing revealed that MCS modulates the expression of osteogenesis-related and inflammation-related factors in hPDLFs primarily through multiple MAPK signaling pathways, including the stress-activated MAPK cascade.

### Yoda1 upregulated the expression of PIEZO1 and osteogenic-related factors and activated the MAPK signaling pathway

Through integrated *in vitro* and *in vivo* experiments combined with transcriptomic analysis, we found that traumatic occlusion suppressed the expression of PIEZO1 and exacerbated the periodontitis, a process that is closely associated with the MAPK signaling pathway. To further investigate the therapeutic potential of PIEZO1 activation, we treated hPDLFs with Yoda1, a specific PIEZO1 agonist, and explored its effects on the MAPK signaling pathway.

**Figure 9:**
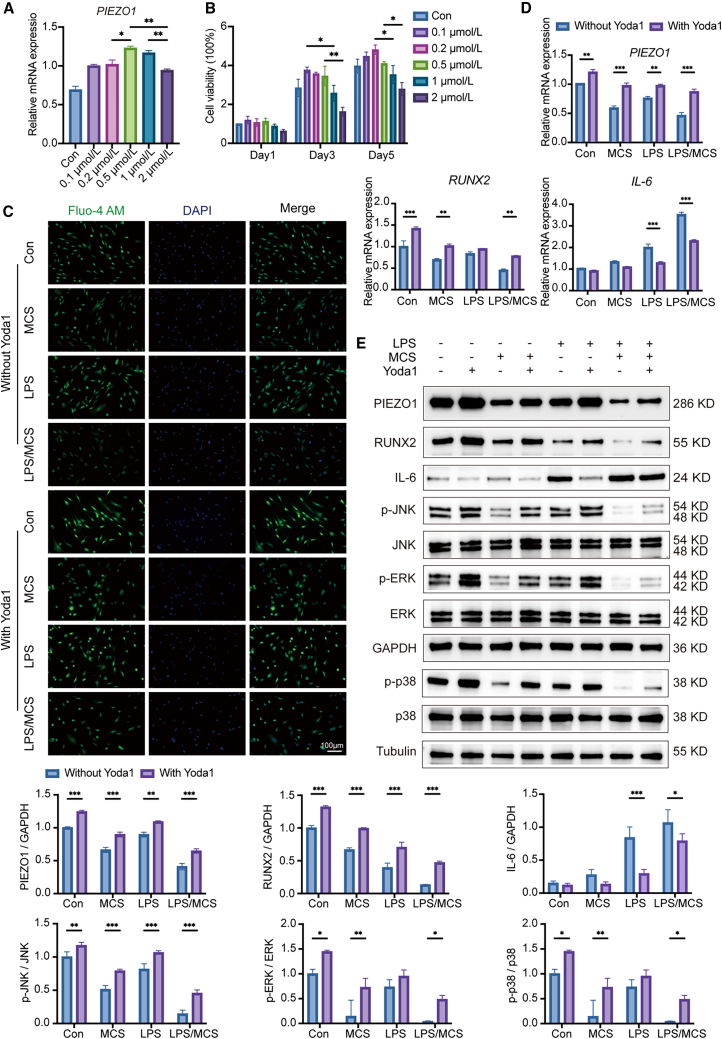
Yoda1 upregulated the expression of PIEZO1 and osteogenic-related factors and activated the MAPK signaling pathway (A) RT-qPCR detected the expression of *PIEZO1*. (B) CCK-8 detected cell proliferation under different concentrations of Yoda1. (C) Fluo-4 AM calcium ion fluorescence staining. Scale bar: 100 μm. (D) RT-qPCR analysis of *PIEZO1*, *RUNX2*, and *IL-6* expression in hPDLFs treated with LPS and mechanical compression stress (MCS), with or without Yoda1. (E) Western blot analysis of PIEZO1, RUNX2, IL-6, p-ERK, ERK, p-JNK, JNK, p-p38, and p38 in the same groups. Quantitative densitometric analysis was performed using ImageJ. Statistical significance was tested with Mann-Whitney U test. ∗*p* < 0.05, ∗∗*p* < 0.01, and ∗∗∗*p* < 0.001; ns, not significant (*p* > 0.05). Error bars represent mean ± SD (*n* = 3 biological replicates).

In the initial phase of these *in vitro* experiments, we evaluated the impact of Yoda1 on hPDLFs at concentrations ranging from 0 to 2 μmol/L. RT-qPCR results revealed a dose-dependent upregulation of *PIEZO1*, with maximal expression observed at 0.5 μmol/L (Figure 9A), while Cell Counting Kit-8 (CCK-8) assays indicated that this concentration did not affect cell proliferation (Figure 9B). Thus, we chose the concentration of 0.5 μmol/L Yoda1 as the optimal concentration.

As depicted in Figure 9C, Fluo-4 Acetoxymethyl ester (Fluo-4 AM) fluorescence staining demonstrated a significant reduction in intracellular Ca^2+^ levels in both the MCS and LPS/MCS groups compared to the Con group, consistent with prior observations. Critically, pharmacological activation of PIEZO1 with Yoda1 robustly reversed this attenuation, eliciting a pronounced rise in fluorescence intensity across experimental groups. These data established Yoda1 as a potent PIEZO1 agonist that potentiates Ca^2+^ influx in the cytoplasm. As shown in Figure 9D, Yoda1 significantly increased the mRNA expression of *PIEZO1* and *RUNX2*, while reducing *IL-6* levels compared to the LPS/MCS group and MCS group. WB analysis (Figure 9E) further confirmed increased protein levels of PIEZO1 and RUNX2, accompanied by enhanced phosphorylation of ERK, JNK, and p38, indicating activation of the MAPK signaling pathway.

### PIEZO1 deficiency inhibited the phosphorylation of MAPK signaling pathway, suppressing osteogenesis and promoting inflammation

**Figure 10:**
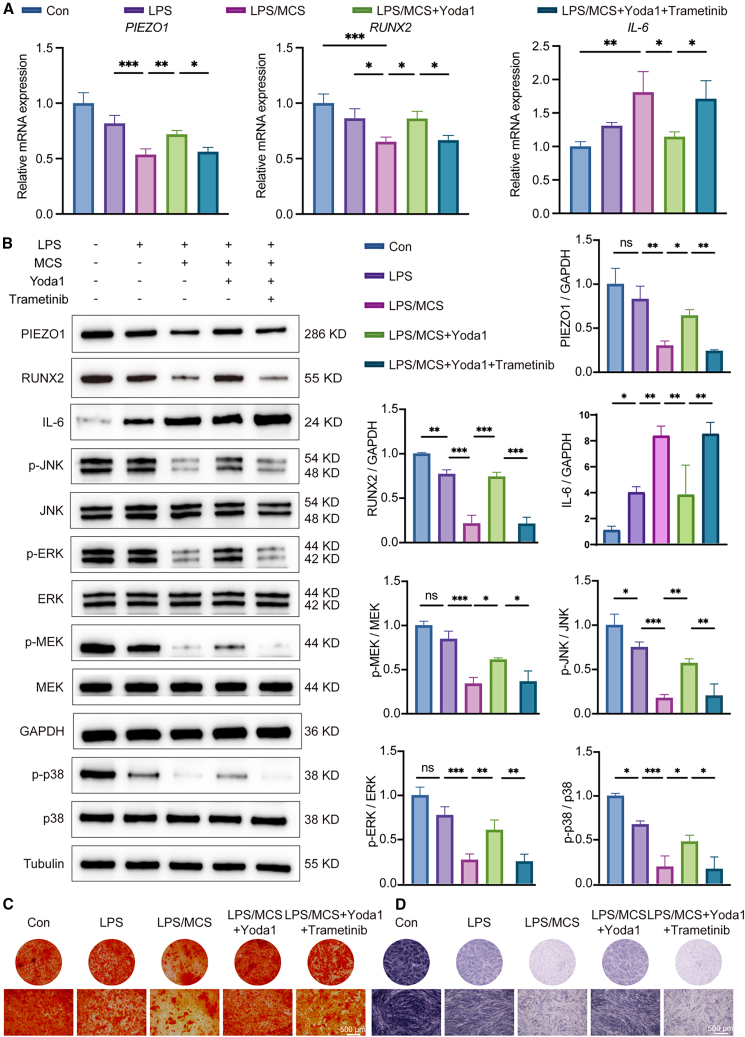
PIEZO1 deficiency inhibited the phosphorylation of MAPK signaling pathway, suppressing osteogenesis and promoting inflammation (A) RT-qPCR analysis of *PIEZO1*, *RUNX2*, and *IL-6* expression in hPDLFs treated with LPS/MCS, Yoda1, and the MEK inhibitor trametinib, alone or in combination. (B) Western blot analysis of PIEZO1, RUNX2, IL-6, p-ERK, ERK, p-JNK, JNK, p-p38, p38, p-MEK, and MEK under the same treatment conditions. (C) ARS staining of hPDLFs with images captured under an inverted microscope. Scale bar: 500 μm. (D) ALP staining of hPDLFs with images captured under an inverted microscope. Scale bar: 500 μm. Quantitative analysis was conducted using ImageJ. Statistical significance was tested with Kruskal-Wallis test followed by Dunn’s *post hoc* test. ∗*p* < 0.05, ∗∗*p* < 0.01, and ∗∗∗*p* < 0.001; ns, not significant (*p* > 0.05). Error bars represent mean ± SD (*n* = 3 biological replicates).

To further assess whether these effects were mediated specifically through MAPK signaling, selective MAPK/ERK Kinase (MEK) inhibitor—trametinib—was applied in combination with Yoda1. As shown in Figure 10A, co-treatment with -trametinib abolished the Yoda1-induced upregulation of *RUNX2* and the suppression of *IL-6*, with mild altering *PIEZO1* expression. WB results (Figure 10B) demonstrated that the activation of MAPK signaling pathway induced by Yoda1 was effectively blocked by trametinib. Consistent with the previous results, Yoda1 restored the expression of PIEZO1 and RUNX2 and suppressed IL-6 under LPS and MCS conditions. However, these effects were significantly attenuated when ERK phosphorylation was pharmacologically inhibited by trametinib, indicating that the ERK branch of MAPK signaling is required for the protective effects of Yoda1. The branch-specific contributions of JNK and p38 remain to be clarified. To further assess osteogenic function, ALP staining (Figure 10C) and ARS staining (Figure 10D) were performed. Yoda1 treatment rescued the LPS- and MCS-induced suppression of ALP activity and reduction in mineralized nodule formation. Conversely, co-treatment with the MEK inhibitor trametinib abrogated Yoda1’s pro-osteogenic effects.

These results suggest that PIEZO1 mediates its osteogenic and anti-inflammatory effects through MAPK signaling pathway and that this pathway is essential for the functional response to PIEZO1 activation under mechanical and inflammatory stress.

**Figure 11:**
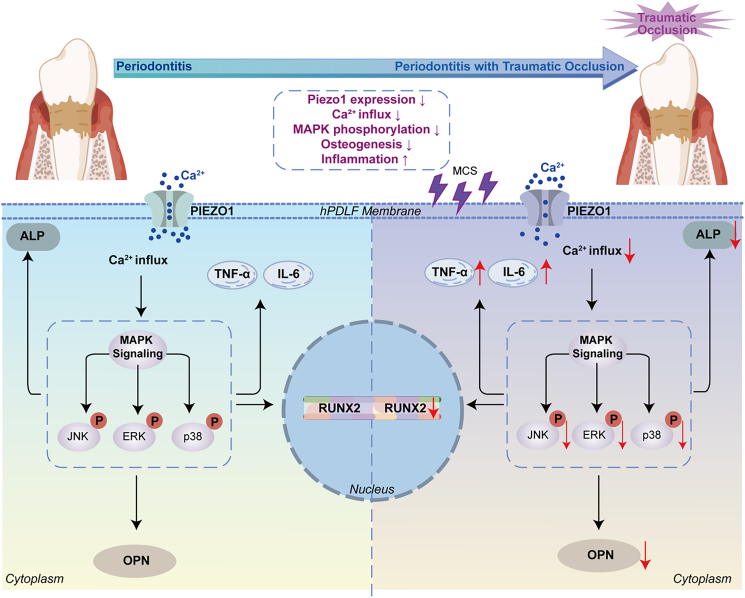
Schematic diagram illustrated the mechanisms by which traumatic occlusion aggravated periodontitis via PIEZO1 and its association with the MAPK signaling pathway This figure was created by Figdraw.

In summary, *in vitro* experiments revealed that traumatic occlusal force suppressed PIEZO1 expression in hPDLFs and attenuated phosphorylation of the MAPK signaling pathway, leading to an enhanced inflammatory response and reduced osteogenic ability (Figure 11).

## Discussion

Physiological occlusal forces dynamically adapt to functional demands by transmitting mechanical forces to periodontal tissues, which undergo adaptive remodeling to maintain homeostasis.^20^^,^^21^ However, excessive or misaligned forces loading disrupts this homeostasis, leading to pathological alterations.^22^ In this study, we first identified the critical role of PIEZO1 in the exacerbation of periodontitis due to traumatic occlusion through *in vivo* assays. To further investigate the underlying mechanism, we explored the involvement of PIEZO1 in decreased osteogenesis of hPDLFs under combined *Porphyromonas gingivalis* LPS and MCS.

*In vivo*, rat models of PD, TO, and PD/TO dual challenges were established over 1, 2, and 4 weeks to evaluate temporal progression. Notably, TO alone only exhibited a detectable bone density reduction at 4 weeks, without significant hard or soft tissue attachment loss. This is consistent with the findings of Fan et al.^23^ that excessive occlusal force does not trigger periodontal disease in the absence of plaque attachment. However, in the presence of periodontitis, TO accelerates disease progression as early as 1 week, with severity worsening over time.

Mechanical force is a well-established trigger of bone remodeling^24^; however, the specific role of occlusal force in the maintenance of alveolar bone mass remains unclear.^25^ It has been demonstrated that the PIEZO1 functioned as a mechanosensor for occlusal forces, and its activation facilitates the repair of alveolar bone loss under conditions of occlusal unloading.^26^ Consistent with this, our data revealed significant downregulation of PIEZO1 and RUNX2 in both TO and PD/TO groups across all time points. We hypothesize that excessive occlusal force may suppress the expression of PIEZO1, thereby accelerating bone resorption. To test this hypothesis, we administered the PIEZO1 agonist Yoda1 into the periodontal pockets of PD/TO rats. Micro-CT analysis along with H&E and Masson’s trichrome staining revealed attenuated periodontal tissue destruction. Additionally, immunohistochemical analysis showed increased expression of the osteogenic marker RUNX2 and decreased expression of the pro-inflammatory cytokine IL-6. Importantly, we also observed enhanced phosphorylation of ERK and JNK, key components of the MAPK pathway, in the periodontal tissues following Yoda1 treatment. These findings indicate that PIEZO1 functionally associates with the MAPK pathway. Modulation of PIEZO1 activity may therefore ameliorate alveolar bone resorption in periodontitis.

As a component of the periodontal tissues, the PDL is a specialized connective tissue that connects the teeth to the alveolar bone. The PDL comprises a heterogeneous cellular population, including fibroblasts, cementoblasts, osteoblasts, and osteoclasts,^27^ with fibroblasts constituting the predominant cell type. To further investigate the mechanisms of human traumatic occlusion on periodontitis, we established an *in vitro* model using hPDLFs.

In our experimental design, hPDLFs were subjected to MCS overloading to simulate traumatic occlusion,^28^ in combination with LPS treatment to induce an inflammatory state.^29^ The results revealed that both MCS overload and LPS stimulation reduced PIEZO1 expression, with the most pronounced inhibition observed under combined stimulation. However, the individual contributions of mechanical versus inflammatory stimulation to PIEZO1 downregulation remain unclear, and further studies are warranted to quantify their respective effects. Notably, the LPS/MCS group exhibited enhanced inflammation, suppressed osteogenesis, and a significant reduction in PIEZO1 expression compared to the LPS group, while Zhu et al.^30^ found that stimulation with high-magnitude mechanical force (0.2 g/cm^2^) activates PIEZO1 of PDL cells, thereby reducing the immunoregulatory capacity and slowing down orthodontic tooth movement. These findings differ from our results, and the discrepancies may be attributed to variations in loading parameters (magnitude and duration) and cell types.

PIEZO1, a mechanosensitive calcium channel crucial in maintaining and regulating bone homeostasis,^31^ was consistently demonstrated to be downregulated in both *in vivo* and *in vitro* models through our experimental series. Besides, the opening of transmembrane channels was inhibited, and the influx of calcium ions was reduced *in vitro* models. To elucidate the molecular mechanisms underlying this phenomenon, we conducted transcriptome sequencing analysis. The results showed that MAPK signaling pathway was significantly enriched. Based on these findings, we hypothesized that the mechanism by which PIEZO1 contributes to the exacerbation of periodontitis by occlusal trauma is related to the MAPK pathway. Activation of PIEZO1 expression may mitigate the exacerbating effect of traumatic occlusion on periodontitis.

The MAPK signaling pathway plays a crucial role in regulating key biological processes, including cell proliferation, osteogenic differentiation, and inflammation.^32^^,^^33^^,^^34^ Meanwhile, the MAPK pathway is closely linked to osteogenic mineralization in osteoblasts, with activation of the MAPK/ERK1/2 pathway promoting osteogenic gene expression and osteoblast proliferation.^35^ The major components of the MAPK signaling pathway, ERK, JNK, and p38, are all associated with cellular responses to mechanical stress.^36^^,^^37^ Additionally, the MAPK signaling pathway is a potential target for inflammatory mediators and plays a crucial role in the pathogenesis of periodontitis.^38^ It had demonstrated that *Porphyromonas gingivalis*-derived LPS modulates the inflammatory response by activating the MAPK signaling pathway in hPDLFs.^39^

Subsequently, to investigate PIEZO1-MAPK crosstalk and whether PIEZO1 activation enhances the osteogenic capacity, we employed Yoda1, a selective PIEZO1 agonist,^40^ in hPDLFs under inflammatory and mechanical stress conditions. The results indicated that, compared to the LPS group, the activation of PIEZO1 channels was inhibited, the influx of calcium ions was reduced, and the expression of PIEZO1 and RUNX2 was elevated, while the levels of p-ERK, p-JNK, and p38 were decreased in the LPS/MCS group. Yoda1 administration reversed this pattern, significantly upregulating PIEZO1, RUNX2, and MAPK phosphorylation (p-ERK, p-JNK, and p38) across all experimental groups. These findings align with our hypothesis that PIEZO1 activation rescues MAPK signaling dysregulation, thereby restoring osteogenic capacity in inflamed hPDLFs. To further validate the mechanistic role of MAPK signaling downstream of PIEZO1, we conducted inhibition experiments using selective MEK/ERK inhibitor—trametinib. These pharmacological inhibition experiments further confirm that the PIEZO1-mediated restoration of osteogenic capacity and suppression of inflammation depend on MAPK signaling, particularly on ERK signaling. The reversal of Yoda1’s effects upon ERK blockade supports a model in which PIEZO1 acts upstream of MAPK to regulate periodontal cellular responses to combined mechanical and inflammatory stimuli. It should be noted that trametinib primarily targets the MEK/ERK axis. While our data suggest potential involvement of other MAPK branches, the specific contributions of JNK and p38 signaling remain to be fully elucidated and warrant further investigation.

Based on the collective findings, we conclude that traumatic occlusion accelerates the early progression of periodontitis, primarily by impairing mechanotransduction and osteogenic function through PIEZO1 downregulation. Mechanical overload reduced the osteogenic potential of hPDLFs under both physiological and inflammatory conditions via suppression of PIEZO1 expression and activation. Importantly, pharmacological activation of PIEZO1 rescued this impairment, restoring mineralization capacity in hPDLFs even in the presence of inflammation. Mechanistically, these effects involved MAPK signaling, as PIEZO1 modulated osteogenic differentiation through ERK activation under mechanical and inflammatory stress, while the branch-specific contributions of JNK and p38 remain to be clarified. Although Yoda1 effectively reversed inflammatory and osteogenic dysfunction in our models, it is an experimental tool compound rather than a clinically viable drug. Therefore, while our findings support the therapeutic relevance of targeting PIEZO1, further investigation using pharmacologically optimized or clinically translatable PIEZO1 modulators is warranted.

### Limitations of the study

Several limitations should be acknowledged. First, the depth of biological insight is constrained by the limited biomarker panel. Specifically, the absence of key markers like IL-1β, MMPs, and COL1A1 hinders a more comprehensive understanding of inflammation subtleties, dynamic and later stages of osteogenesis. Second, although Yoda1 restored phosphorylation of ERK, JNK, and p38, our inhibitor experiments relied solely on trametinib, which selectively blocks the MEK/ERK pathway. Thus, while our data establish a requirement for ERK signaling, the branch-specific contributions of JNK and p38 remain unresolved and require further study. Finally, the absence of PIEZO1 loss-of-function validation (e.g., knockdown or knockout models) limits the causal confirmation of its role. Future studies employing genetic models, advanced biomechanical simulations, and long-term *in vivo* validation are warranted to further elucidate the PIEZO1-MAPK axis and assess its translational applicability.

## Resource availability

### Lead contact

Requests for further information and resources should be directed to and will be fulfilled by the lead contact, Yun He (heyundaidai@163.com).

### Materials availability

This study did not generate new unique reagents.

### Data and code availability

• RNA-seq data have been deposited at NCBI Sequence Read Archive (SRA): PRJNA1231064 (http://www.ncbi.nlm.nih.gov/bioproject/1231064) and are publicly available.
• This paper does not report original code.
• Any additional information required to reanalyze the data reported in this paper is available from the lead contact upon request.

## Acknowledgments

This work was funded by the Special Project for Local Science and Technology Development Guided by the Central Government of Sichuan Province (2024ZYD0058), Luzhou Science and Special Projected Technology Program (2023SYF116 and 2024RCX210), Sichuan Medical Association Program (2024TG03), and Scientific Research Foundation of Southwest Medical University (2021ZKMS018, 2023ZD002, and 2024KQZX05).

## Author contributions

Conceptualization, B.X.; methodology, X.W. and B.X.; investigation, Y.G.; writing – original draft, X.W. and B.X.; writing—review and editing, H.W. and X.H.; funding acquisition, J.C. and Y.H.; resources, J.C. and Y.H.; supervision, Y.H.

## Declaration of interests

The authors declare no competing interests.

## Declaration of generative AI and AI-assisted technologies in the writing process

The authors did not use AI-assisted technologies.

## STAR★Methods

### Key resources table

**Table:** 

REAGENT or RESOURCE	SOURCE	IDENTIFIER
**Antibodies**		

anti-PIEZO1 primary antibodies	Novus Biologicals, Shanghai, China	Cat# NBP1-78537AF350; RRID:AB_3240030
anti-RUNX2 primary antibodies	Abcam, Cambridge, UK	Cat#ab92336; RRID:AB_2049267
anti-OPN primary antibodies	Abcam, Cambridge, UK	Cat#ab218237; RRID:AB_2732079
anti-phospho-ERK1/2 primary antibodies	Huabio, Hangzhou, China	Cat#ET1603-22; RRID:AB_3069673
anti-ERK1/2 primary antibodies	Huabio, Hangzhou, China	Cat#ET1601-29; RRID:AB_3069611
anti-phospho-JNK 1/2/3 primary antibodies	Huabio, Hangzhou, China	Cat#ET1609-42; RRID:AB_3069852
anti-JNK 1/2/3 primary antibodies	Huabio, Hangzhou, China	Cat#ET1601-28; RRID:AB_3069610
anti-phospho-p38 primary antibodies	Huabio, Hangzhou, China	Cat#HA722657; RRID:AB_3069528
anti-p38 primary antibodies	Huabio, Hangzhou, China	Cat#ET1702-65; RRID:AB_3073466
anti-phospho-MEK primary antibodies	Bio-swamp, Wuhan, China	Cat#PAB56110
anti-MEK primary antibodies	Bioswamp, Wuhan, China	Cat#PAB42340
anti-IL-6 primary antibodies	Bioswamp, Wuhan, China	Cat#MAB48201
anti-Tubulin primary antibodies	Bioswamp, Wuhan, China	Cat#MAB50882
anti-GAPDH primary antibodies	Bioss, Hangzhou, China	Cat#bs-41373R
horseradish peroxidase (HRP)-conjugated goat anti-rabbit secondary antibody	Bioswamp, Wuhan, China	Cat#SAB48169

**Biological samples**		

Sprague-Dawley (SD) rats	a specific pathogen-free (SPF) facility at the Experimental Animal Center of Southwest Medical University	N/A
human periodontal ligament fibroblasts (hPDLFs)	healthy premolars extracted for orthodontic treatment at the Affiliated Stomatological Hospital, Southwest Medical University	N/A

**Chemicals, peptides, and recombinant proteins**		

LPS-PG Ultrapure	Invivogen, San Diego, USA	Cat#tlrl-ppglps
Yoda1	MedChemExpress, New Jersey, USA	Cat#HY-18723
Trametinib	TargetMol, Shanghai, China	Cat#871700-17-3

**Critical commercial assays**		

RNA sequencing and differentially expressed genes analysis	Shanghai Majorbio Bio-pharm Biotechnology Co., Ltd. (Shanghai, China).	N/A

**Deposited data**		

The RNA-seq datasets	NCBI Sequence Read Archive (SRA)	ID: PRJNA1231064

**Experimental models: Organisms/strains**		

Periodontitis (PD) rats, Traumatic Occlusion (TO) ratsPD combined with TO (PD/TO) rats	Sprague-Dawley (SD) rats	N/A

**Oligonucleotides**		

OPN Forward5’-CCAAGTAAGTCCAACGAAAG-3’ Reverse5’-GGTGATGTCCTCGTCTGTA-3’	Sangon, Shanghai, China	N/A
ALP Forward5′-GCCGCCCGCTTTAACC-3′ Reverse5′-CTCCCACTGACTTCCCTGCTT-3′	Sangon, Shanghai, China	N/A
RUNX2 Forward5′-CCCAGTATGAGAGTAGGTGTCC-3′ Reverse5′-GGGTAAGACTGGTCATAGGACC-3′	Sangon, Shanghai, China	N/A
IL-6 Forward5′-CTCTTCTGCCTGCACTTTG-3′ Reverse5′-ATGGGCTACAGGCTTGTCACTC-3′	Sangon, Shanghai, China	N/A
TNF Forward5′-AGACAGCCACTCACCTCTTCAG-3′ Reverse5′-TTCTGCCAGTGCCTCTTTGCT-3′	Sangon, Shanghai, China	N/A
PIEZO1 Forward5′-CCTGGAGAAGACTGACGGCTAC-3′ Reverse5′-ATGCTCCTTGGATGGTGAGTCC-3′	Sangon, Shanghai, China	N/A
GAPDH Forward5′-CAATGACCCCTTCATTGACC-3′ Reverse5′-GACAAGCTTCCCGTTCTCAG-3′	Sangon, Shanghai, China	N/A

**Software and algorithms**		

ImageJ software	National Institutes of Health,NIH	N/A
GraphPad Prism 9.0	(GraphPad Software, San Diego, CA, USA).	N/A

### Experimental model and study participant details

#### Animal model establishment

All rats were housed in a specific pathogen-free (SPF) facility at the Experimental Animal Center of Southwest Medical University. The animal room was maintained at 22 ± 2 °C with 50 ± 10% relative humidity. Before the experiments began, six-week-old male Sprague-Dawley (SD) rats underwent a 7-day acclimatization period in the laboratory environment, they had free access to food and water throughout the study. To minimize pain during model establishment, local anesthesia and inhalation anesthesia were administered prior to all procedures. All experimental procedures were conducted in accordance with the guidelines approved by the Ethics Committee of Southwest Medical University (Approval No: 20221116-031).

##### Establishment of PD/TO rat model

A total of 72 Sprague-Dawley (SD) rats were randomly assigned to four experimental groups: (1) Control (Con): Rats were maintained under standard conditions without any intervention. (2) Periodontitis (PD): Periodontitis was induced by ligating the bilateral maxillary first molars (M1). A 0.2 mm ligation wire was inserted into the interdental space between M1 and M2, looped around the cervical region of M1 from the buccal side, and knotted on the mesial surface. (3) Traumatic Occlusion (TO): a 1 mm-thick layer of composite resin was bonded to the occlusal surface of the bilateral M1 to simulate traumatic occlusion. (4) PD combined with TO (PD/TO): Periodontitis was first induced as described for the PD group. Four weeks later, traumatic occlusion was established on the same M1 teeth. Ligatures and resin were examined every other day under brief inhalation anesthesia, resin was replenished when its height above the occlusal surface was less than 1 mm, and any dislodged ligatures and resin were promptly replaced to ensure experimental consistency. Tissue samples from each group were collected at 1, 2, and 4 weeks following the induction of traumatic occlusion. Rats were euthanized under anesthesia by intraperitoneal injection of pentobarbital sodium solution (150 mg/kg). Maxillary specimens, including bone, teeth, periodontal ligament, and gingival tissues, were harvested for micro-CT, histological and immunofluorescent analyses.

##### Yoda1 treatment in the PD/TO rat model

A total of 48 male SD rats were used, based on the previously described four experimental groups: Con, TO, PD, and PD/TO. Each group was further divided into Yoda1-treated and untreated subgroups (n = 6 per group), resulting in eight experimental groups.

In the Yoda1-treated groups, Yoda1 (10 μM, 20 μL in 10% DMSO/PBS) was locally injected into the buccal gingiva adjacent to the M1 immediately after TO modeling, and then administered every other day for two weeks.^13^ Control animals received equal volumes of vehicle solution under the same protocol.

During the experimental period, the integrity of ligatures and resin was checked every 48 hours under brief inhalation anesthesia, and adjustments were made as needed. At the end of the treatment, all animals were humanely euthanized under deep anesthesia via intraperitoneal injection of pentobarbital sodium (150 mg/kg). Maxillary tissues were harvested for micro-CT, histological and immunofluorescent analyses.

#### Cell culture and identification

Periodontal ligament tissue was collected from healthy premolars extracted for orthodontic treatment at the Affiliated Stomatological Hospital, Southwest Medical University. Informed consent was obtained from 20 donors (aged 12–16 years) and their legal guardians. This study was approved by the Institutional Ethics Committee of the Affiliated Stomatological Hospital, Southwest Medical University (certificate number: 20211209004) and was conducted in accordance with the Declaration of Helsinki and its later amendments or comparable ethicalstandards. The PDL was scraped from the middle third of root surface and minced into small fragments. HPDLFs were isolated and cultured with α-MEM media, supplemented with 10% fetal bovine serum (FBS), 100 U/mL penicillin-G, and 100 μg/mL streptomycin sulfate. The cells were incubated at 37°C with 5% CO_2_. Upon reaching 90% confluence, hPDLFs were passaged, and cells from passages 3 to 5 were used in subsequent experiments.

Alizarin Red and Oil Red O staining were performed to assess the multidirectional differentiation potential of the cells. Cell phenotypes were identified through immunofluorescence staining of vimentin and keratin, and further analyzed by flow cytometry analysis.

For the first part of the subsequent experiment, hPDLFs were divided into four groups: (1) control (Con) group: hPDLFs cultured under standard conditions without intervention, serving as the negative control. (2) mechanical compressive stress (MCS) group: hPDLFs subjected to MCS loading for 12 h. (3) lipopolysaccharide (LPS) group: hPDLFs treated with 1 μg/mL LPS derived from *Porphyromonas gingivalis* (InvivoGen, San Diego, CA, USA) for 12 h. (4) LPS/MCS group: hPDLFs were simultaneously treated with LPS and subjected to MCS for 12 h.

For the second part of the *in vitro* experiment, hPDLFs were divided into two main groups: (1) Non-Yoda1 group: hPDLFs without Yoda1 treatment, further subdivided into the four subgroups described above; (2) Yoda1 group: hPDLFs treated with 0.5 μM Yoda1 dissolved in dimethyl sulfoxide (DMSO) and co-incubated with LPS and/or MCS for the same 12-hour period, also subdivided into the same four subgroups. The same final concentration of DMSO (0.1%) was added to all control and experimental groups.

For the third part of the *in vitro* experiment, hPDLFs were pre-treated with 200 nM Trametinib (in DMSO) for 24 hours. Subsequently, Yoda1 was added and co-incubated with LPS and MCS. All control and experimental groups received the same final concentration of DMSO (0.1%).

3 It is important to note a limitation of our study: only male rats were used. This choice was made primarily to avoid the potential confounding effects of hormonal fluctuations associated with the estrous cycle in females, which can influence inflammatory responses and bone metabolism. While this provides internal consistency for our results, it simultaneously limits the direct generalizability of our findings to females. Future studies are warranted to investigate whether similar mechanisms and therapeutic effects of Yoda1 are observed in female animal models and to explore the influence of sex as a biological variable on the progression of periodontitis exacerbated by traumatic occlusion.

### Method details

#### Micro-CT analysis

Three samples were randomly selected from each group and scanned using nanoVoxel-1000 to obtain Micro-CT data. The vertical distance from the cementoenamel junction to the alveolar bone crest (CEJ-ABC) was recorded in each sample.

#### *In vivo* histology and osteoclast analysis

Tissue samples were fixed in 10% neutral-buffered paraformaldehyde for 24 hours, followed by decalcification in 10% EDTA solution for 4 weeks at room temperature with regular solution changes. After decalcification, samples were embedded in paraffin and sectioned into 4-μm-thick slices for staining.

For histomorphological assessment, hematoxylin and eosin (H&E) staining was performed to evaluate overall tissue structure and inflammatory cell infiltration. Masson’s trichrome staining was used to assess collagen content and fiber organization in the periodontal ligament.

Tartrate-resistant acid phosphatase (TRAP) staining was conducted to evaluate osteoclast activity and quantify bone resorption within the region of interest (ROI).

For protein-level analysis, IHC was carried out to detect the expression of PIEZO1, RUNX2, and interleukin-6 (IL-6) in the ROI using specific primary antibodies and a standard DAB chromogenic detection system. Additionally, IHF staining was performed on deparaffinized sections to visualize the localization and relative expression of target proteins (including PIEZO1, p-JNK, ERK and p-ERK), using fluorophore-conjugated secondary antibodies and DAPI nuclear counterstaining.

All stained sections were imaged using a light microscope (Olympus, Tokyo, Japan) or fluorescence microscope (Nikon, Tokyo, Japan), and quantitative analyses were performed using ImageJ software.

#### MCS loading

The cell density was adjusted to 1 × 10^5^ cells per loading plate. MCS was applied on cells using a four-point bending mechanical force loading device (Patent No: CN 2534576Y, West China School of Stomatology, Sichuan University) when cells reached 80% confluence. A MCS of 4000 μstrain was applied, with the parameters set to a displacement of 2.2 mm and a frequency of 0.5 Hz [20]. The loading time was set as 0, 3, 6, 12, and 24 hours. The expression of PIEZO1 was detected by quantitative real-time PCR (RT-qPCR) to determine the optimal loading time.

BCIP/NBT Alkaline Phosphatase Chromogenic Kit (Beyotime, Shanghai, China) following the manufacturer's instructions. The ALP staining results were first photographed and subsequently examined under an inverted microscope (Olympus IX71, Tokyo, Japan).

#### Quantitative real-time PCR (RT-qPCR)

Total RNA was extracted from hPDLFs in each group using an RNA extraction kit (Bioteke, Beijing, China). After reverse transcription using HiScript III ReverTra Ace qPCR Master Mix (TOYOBO, Chuo-ku, Japan), the cDNA samples were amplified and quantified using QuantiNova SYBR Green PCR Master Mix (TOYOBO, Chuo-ku, Japan) on a CFX Maestro™ software (Bio-Rad, California, USA). The target genes and corresponding primer sequences are presented in below Table.

**Table 1:** Primer sequences of genes for RT-qPCR

Gene	Primer	
OPN	forward	5′-CCAAGTAAGTCCAACGAAAG-3′
	reverse	5′-GGTGATGTCCTCGTCTGTA-3′
ALP	forward	5′-GCCGCCCGCTTTAACC-3′
	reverse	5′-CTCCCACTGACTTCCCTGCTT-3′
RUNX2	forward	5′-CCCAGTATGAGAGTAGGTGTCC-3′
	reverse	5′-GGGTAAGACTGGTCATAGGACC-3′
IL-6	forward	5′-CTCTTCTGCCTGCACTTTG-3′
	reverse	5′-ATGGGCTACAGGCTTGTCACTC-3′
TNF	forward	5′-AGACAGCCACTCACCTCTTCAG-3′
	reverse	5′-TTCTGCCAGTGCCTCTTTGCT-3′
PIEZO1	forward	5′-CCTGGAGAAGACTGACGGCTAC-3′
	reverse	5′-ATGCTCCTTGGATGGTGAGTCC-3′
GAPDH	forward	5′-CAATGACCCCTTCATTGACC-3′
	reverse	5′-GACAAGCTTCCCGTTCTCAG-3′

#### RNA sequencing and differentially expressed genes analysis

Total RNA was extracted from hPDLFs across four groups using TRIzol Reagent (Invitrogen, Carlsbad, CA, USA). RNA quality was assessed using the 5300 Bioanalyzer (Agilent Technologies, Santa Clara, USA) and quantified with the NanoDrop-2000 (Thermo Fisher Scientific, Wilmington, DE, USA). Libraries were then constructed using the TruSeq Stranded mRNA LT Sample Prep Kit (Illumina, San Diego, CA, USA) according to the manufacturer's instructions. Transcriptome sequencing and bioinformatics analysis were conducted by Shanghai Majorbio Bio-pharm Biotechnology Co., Ltd. (Shanghai, China). The libraries were sequenced on an Illumina HiSeq X Ten platform, generating 150-bp paired-end reads. Raw data were preprocessed using Trimmomatic to remove low-quality reads, resulting in clean reads. These clean reads were mapped to the Homo sapiens reference genome (GRCm38) reference genome using HISAT2. Gene expression levels were quantified as fragments per kilobase of transcript per million mapped reads (FPKM) using Cufflinks, and the read counts of each gene were obtained using HTSeq-count. Differential expression analysis was performed using the DESeq2 R package with adjusted p-value < 0.05 and |log2(fold change)| ≥ 2 as significance thresholds. Hierarchical clustering of differentially expressed genes (DEGs) was conducted to illustrate the expression patterns across different groups. Gene Ontology (GO) enrichment and Kyoto Encyclopedia of Genes and Genomes (KEGG) pathway enrichment analyses of DEGs were performed using the clusterProfiler R package, with statistical significance determined by hypergeometric distribution testing (*p* < 0.05).

#### Western blot (WB)

Total protein was collected from each group using a total protein extraction kit (Keygen Biotech, Nanjing, China). Protein concentrations were quantified, and 20 μg proteins per sample were electrophoresed using 10% SDS-PAGE gels and transferred onto PVDF membranes (Biosharp, Hefei, China). The Multicolor Prestained Protein Ladder (10–250 kDa, WJ03; Enzyme, Shanghai, China) was used as a molecular weight marker in all experiments. After blocking, the membranes were incubated overnight with the following rabbit primary antibodies: anti-PIEZO1 (1:1000, orb637063; Biorbyt, Cambridge, UK), anti-RUNX2 (1:1000, ab92336; Abcam, Cambridge, UK), anti-OPN (1:1000, ab218237; Abcam, Cambridge, UK), anti-phospho-ERK1/2 (1:5000, ET1603-22; Huabio, Hangzhou, China), anti-ERK1/2 (1:5000, ET1601-29; Huabio, Hangzhou, China), anti-phospho-JNK 1/2/3 (1:2000, ET1609-42; Huabio, Hangzhou, China), anti-JNK 1/2/3 (1:2000, ET1601-28; Huabio, Hangzhou, China), anti-phospho-p38 (1:2000, HA722657; Huabio, Hangzhou, China), anti-p38 (1:2000, ET1702-65; Huabio, Hangzhou, China), anti-phospho-MEK (1:1000, PAB56110; Bio-swamp, Wuhan, China), anti-MEK (1:1000, PAB42340; Bio-swamp, Wuhan, China), anti-IL-6 (1:1000, MAB48201; Bio-swamp, Wuhan, China), anti-Tubulin (1:10000, MAB50882; Bio-swamp, Wuhan, China) and anti-GAPDH (1:1000, bs-41373R; Bioss, Hangzhou, China). After washing, membranes were incubated with a horseradish peroxidase (HRP)-conjugated goat anti-rabbit secondary antibody (1:5000; Elabscience, Wuhan, China) for 1 hour at room temperature. Protein bands on the PVDF membranes were visualized using ECL luminescent solution (Affinitia Biosciences, Cincinnati, OH, USA) and the iBrightCL 1000 imaging system (Thermo Fisher Scientific, Massachusetts, MA, USA). For all experiments, proteins of interest and their corresponding loading controls were developed on membranes originating from the same gel.

#### Immunocytofluorescence (ICF) staining

IF staining was performed to analyze the relative expression and localization of PIEZO1 proteins in hPDLFs. After treatment, cells in each group were transferred to a 100-mm plate and washed twice with phosphate-buffered saline (PBS). 4% paraformaldehyde (PFA) was added to fix cells for 30 min. Followed by permeabilization with 0.5% Triton X-100 for 20 minutes. After blocking, cells were incubated overnight at 4°C with a rabbit anti-human PIEZO1 primary antibody (1:200; Biorbyt, Cambridge, UK). The next day, cells were incubated with a fluorescein isothiocyanate (FITC)-conjugated goat anti-rabbit IgG secondary antibody (1:200; Elabscience, Wuhan, China) in the dark and incubated at 37°C for 1 h. Nuclei were counterstained with 4′,6-diamidino-2-phenylindole (DAPI) for 5 minutes in the dark. Images were collected timely under an inverted fluorescence microscope.

### Quantification and statistical analysis

Data were presented as mean ± standard deviation (SD) from at least three independent experiments. Given the small sample size (n = 3), non-parametric tests were used for statistical analysis. For comparisons between two groups, the Mann–Whitney U test was applied. For multiple group comparisons, the Kruskal–Wallis test followed by Dunn’s post hoc test was performed. All analyses were conducted using GraphPad Prism 9.0 (GraphPad Software, San Diego, CA, USA). A p-value < 0.05 was considered statistically significant. Significance levels were denoted as follows:N.S. = not significant, ∗p < 0.05, ∗∗p < 0.01, ∗∗∗p < 0.001; ns = not significant (p > 0.05).

### Additional resources

All animal experimental procedures were conducted in accordance with the guidelines approved by the Ethics Committee of Southwest Medical University (Approval No: 20221116-031).

This study was approved by the Institutional Ethics Committee of the Affiliated Stomatological Hospital, Southwest Medical University (certificate number: 20211209004) .

RNA-seq data have been deposited at NCBI Sequence Read Archive (SRA) under BioProject ID: PRJNA1231064 (http://www.ncbi.nlm.nih.gov/bioproject/1231064) and are publicly available.
