# Supplementary material for: Agonism of PIEZO1 prevents aggravated periodontitis with traumatic occlusion via MAPK signaling pathway
Source: iScience. 2025 Oct 8;28(11):113688. doi: 10.1016/j.isci.2025.113688 (PMC12605257; doi:10.1016/j.isci.2025.113688)
Supplement: Document S1. Figures S1–S3 [file mmc1.pdf]

iScience, Volume *28*

Supplemental information

Agonism of PIEZO1 prevents aggravated periodontitis with traumatic occlusion via MAPK signaling pathway

Xia Wang, Binqing Xie, Ye Guo, Haiyin Wan, Xianyi He, Junliang Chen, and Yun He

Raw data of WB in figure S1, related to Figure 7. Raw data of WB in figure S2, related to Figure 9. Raw data of WB in figure S3, related to Figure 10.

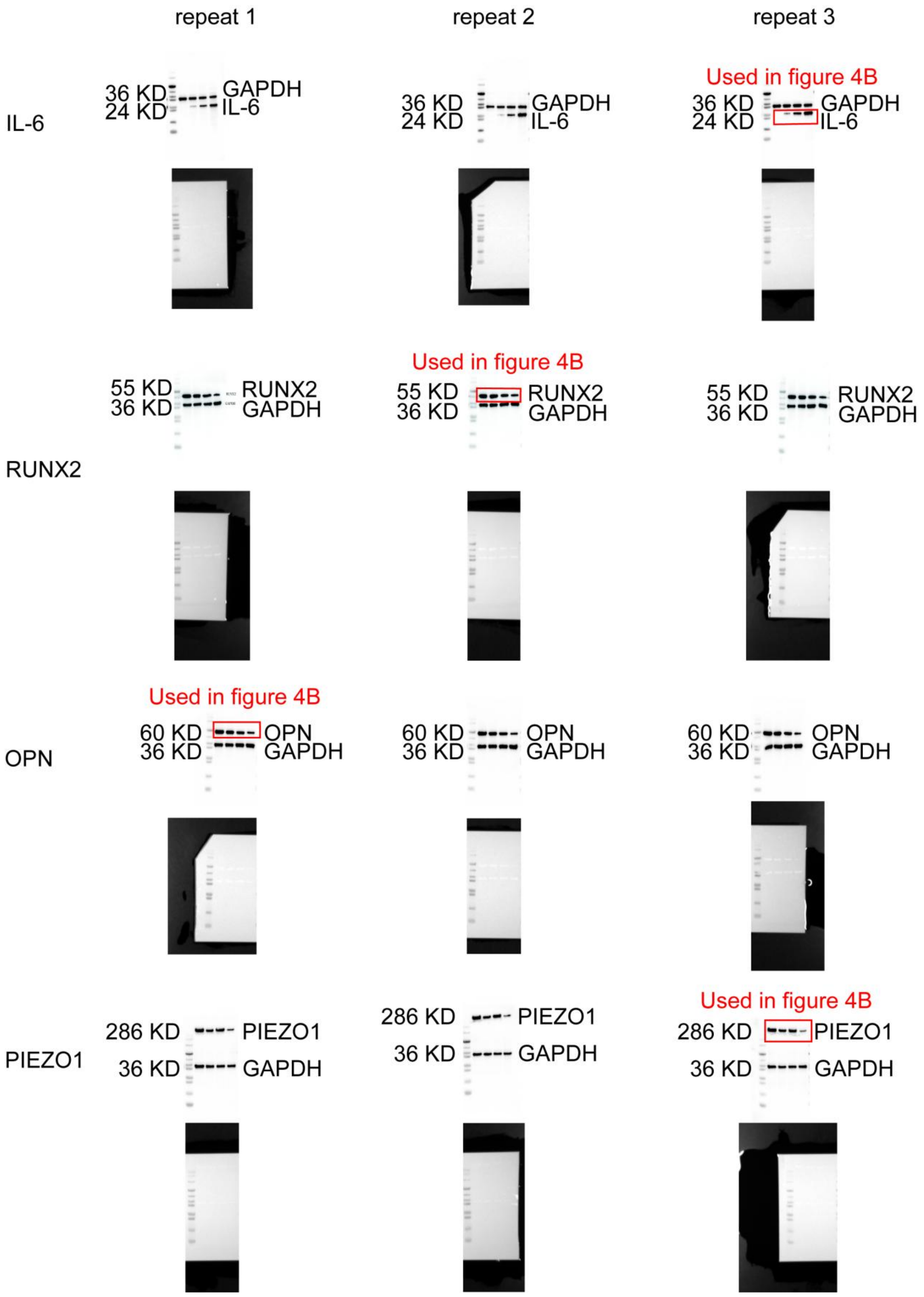

Figure S1. Raw data of WB in figure 7.

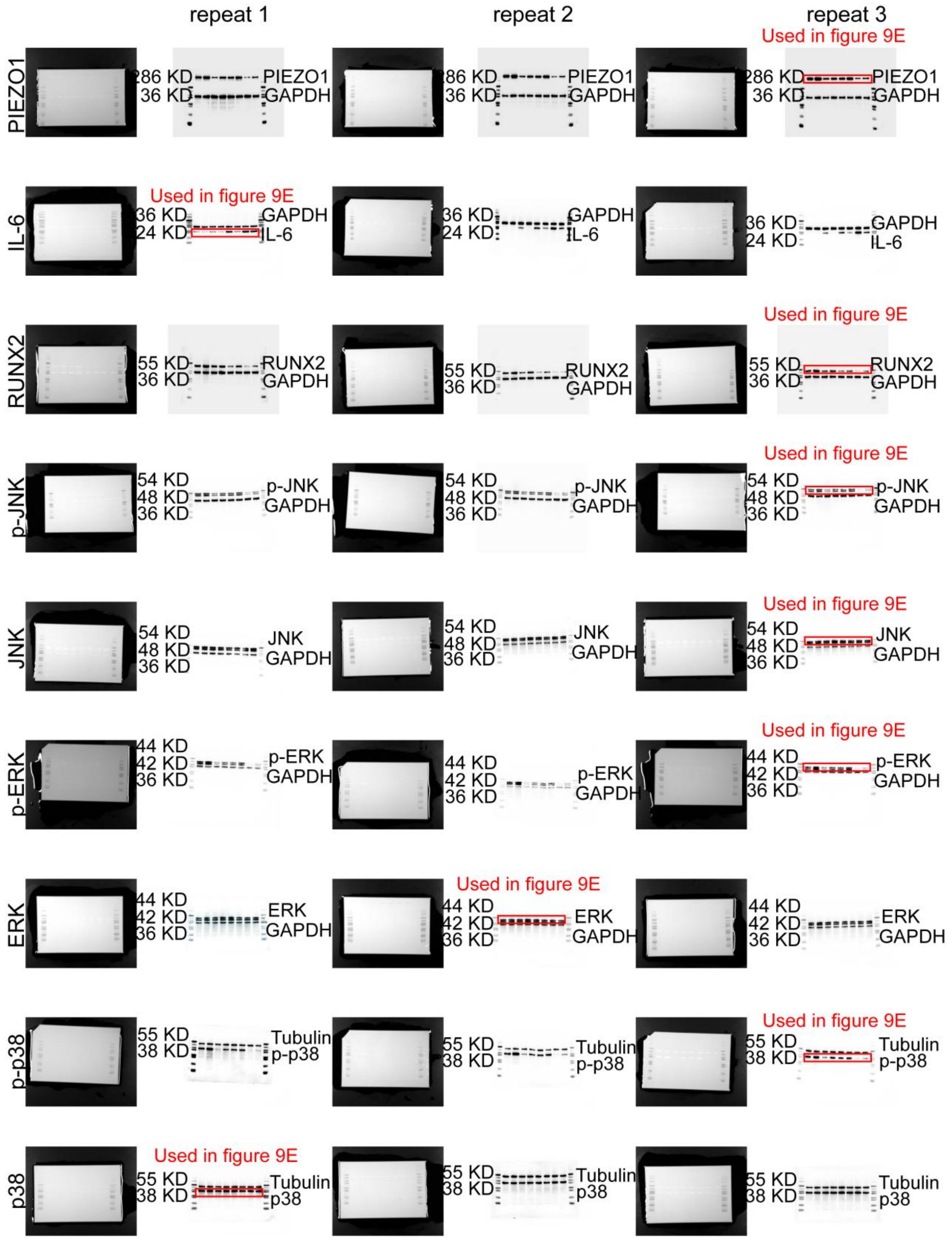

Figure S2. Raw data of WB in figure 9.

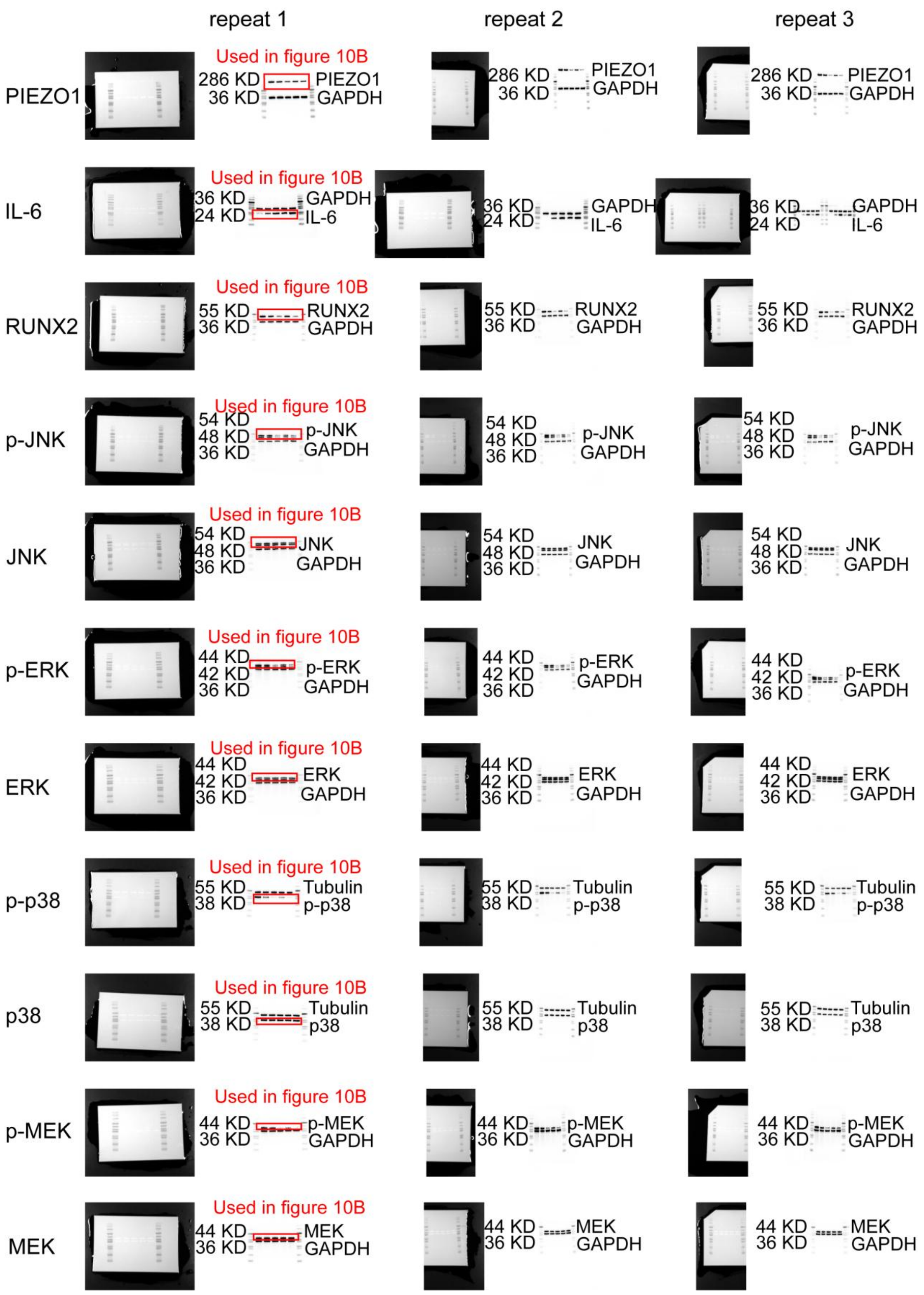

Figure S3. Raw data of WB in figure 10.
